# Treatments for Eating Disorders in People with Autism Spectrum Disorder: A Scoping Review

**DOI:** 10.3390/pediatric17020035

**Published:** 2025-03-12

**Authors:** Rachele Simeon, Giovanni Galeoto, Serena Cracolici, Francescaroberta Panuccio, Anna Berardi

**Affiliations:** 1 Department of Neuroscience, Rehabilitation, Ophthalmology, Genetics, Maternal and Child Health (DINOGMI), University of Genoa, 16132 Genoa, Italy; rachele.simeon@edu.unige.it; 2 Department of Human Neurosciences, Sapienza University of Rome, 00185 Rome, Italy; giovanni.galeoto@uniroma1.it (G.G.); francescaroberta.panuccio@uniroma1.it (F.P.); 3 School of Occupational Therapy, Sapienza University of Rome, 00185 Rome, Italy; cracolici.1895289@studenti.uniroma1.it

**Keywords:** autism, autism spectrum disorder, eating disorders, food selectivity, food neophobia, rumination, anorexia nervosa, pica, obesity, AFRID, treatments, occupational therapy, systematic review

## Abstract

Background: This scoping review aims to synthesize existing evidence on non-pharmacological interventions for managing food selectivity in individuals with autism spectrum disorder (ASD). Specifically, it explores sensory, behavioral, and environmental factors influencing intervention outcomes and examines the role of occupational therapists (OTs) within multidisciplinary teams. Methods: A search was conducted across MEDLINE, EBSCO, Web of Science, OTseeker, and SCOPUS from August 2023 to October 2023. Only experimental studies published in English were included, focusing on behavioral treatments and/or occupational therapy interventions. Results: A total of 1618 studies were identified. After removing duplicates (170 records), 259 full-text articles were assessed for eligibility, resulting in 61 studies included for qualitative synthesis. Conclusions: The findings highlight a wide range of interventions, yet methodological inconsistencies and small sample sizes limit the strength of the evidence. While occupational therapists play an increasing role in feeding interventions, their specific impact remains underexplored. Future research should focus on larger, well-designed studies with standardized outcome measures to better define the effectiveness of interventions and the role of OTs within multidisciplinary teams.

## 1. Introduction

Autism spectrum disorder (ASD) is classified in the Diagnostic and Statistical Manual of Mental Disorders (DSM-5) as a chronic neurodevelopmental disorder with a multifactorial etiology encompassing genetic, neurobiological, and environmental determinants [1]. It is characterized by persistent deficits in social communication and interaction across multiple contexts, along with restricted, repetitive patterns of behavior, interests, or activities. These core symptoms, including impaired reciprocal social interaction and highly stereotyped interests, significantly impact daily functioning. Symptoms typically emerge in early childhood, becoming apparent when social demands exceed developmental capacities, and their severity varies depending on context [2]. Recent studies highlight a rising global prevalence of ASD, with estimates suggesting that approximately 1 in 100 children worldwide receive a diagnosis [3].

Common comorbidities include cognitive impairments, hyperactivity, and difficulties in affective and sensory regulation. Sensory sensitivities—hypo- or hyper-sensitivity to stimuli—are particularly prevalent, often contributing to altered adaptive responses, including maladaptive eating behaviors [1,4]. For instance, hyper-sensitivity to taste, texture, or temperature may lead to the rejection of foods, while hypo-sensitivity may manifest as the ingestion of non-food items. Feeding difficulties affect up to 75% of children with ASD, with food selectivity, also known as selective eating disorder SED) [1,5] being the most common presentation [6]. This condition frequently overlaps with avoidant/restrictive food intake disorder (ARFID), a condition characterized by a restriction or avoidance of food intake that may lead to nutritional deficiencies and impair physical health [6]. Maladaptive eating behaviors, such as SED or anorexia nervosa [7,8], obesity, repeated ingestion of non-food products (PICA), restrictive eating avoidance disorder (AFRID), rumination and food neophobia [9], significantly compromise both physical health and psychosocial functioning. These disturbances disrupt family routines, elevate caregiver stress, and limit social participation, creating challenges that affect multiple domains of life. Among these, food selectivity stands out as one of the most pervasive and difficult symptoms to manage, with profound implications for nutritional status, quality of life, and overall family dynamics. While sensory sensitivities to taste and texture are recognized as contributing factors, they are not the sole determinants. Other influences, such as rigid adherence to routines or aversive experiences (e.g., choking or vomiting), may also play a role. Furthermore, diagnostic frameworks developed for the general population are often challenging to apply to individuals with severe cognitive impairments and high support needs, leading to inconsistencies in diagnosis and treatment approaches. While diagnostic criteria and therapeutic strategies for food selectivity in ASD remain inconsistent, the primary gap in the literature concerns the lack of clarity regarding the specific roles of healthcare professionals in managing these feeding difficulties. Despite the clinical significance of food selectivity in ASD, research remains fragmented, with significant gaps in understanding professional roles and intervention strategies. While existing studies have explored various interventions, there is a lack of clarity regarding the specific professional roles involved in managing food selectivity in children with ASD. The contribution of different specialists—such as occupational therapists, nutritionists, psychologists, and speech therapists—remains poorly defined, leading to inconsistencies in multidisciplinary approaches.

Unlike other aspects of ASD-related feeding difficulties, treatment standardization regarding type and duration is not a primary gap in the literature. However, the absence of a clear framework outlining the roles and responsibilities of healthcare professionals hinders the development of integrated, evidence-based interventions. A scoping review is therefore needed to map the existing evidence, clarify the involvement of different professionals, and provide guidance on how multidisciplinary teams can optimize treatment strategies for food selectivity in ASD. Pharmacological treatments, such as selective serotonin reuptake inhibitors (SSRIs) and antipsychotics, have been explored in ASD-related feeding difficulties, but their efficacy remains uncertain. Studies suggest that while these medications may help with comorbid anxiety or obsessive–compulsive behaviors, they do not directly target food selectivity and often come with significant side effects, such as weight gain and metabolic disturbances. In contrast, behavioral and sensory-based interventions address the underlying mechanisms of food selectivity and have shown promising results in modifying maladaptive eating behaviors. Given these considerations, this review focuses on non-pharmacological interventions as a more targeted and sustainable approach to managing food selectivity in ASD [3]. These findings align with the growing recognition of the need for holistic, individualized approaches that address behavioral and contextual factors influencing feeding difficulties [10]. The majority of programs are based on behavioral, sensory, and nutritional approaches. Behavioral therapies, such as Applied Behavior Analysis (ABA), use positive reinforcement and escape extinction strategies to reduce food avoidance. Sensory interventions focus on gradual desensitization to tastes and textures, while nutritional approaches involve nutrition education and diet customization [11].

The involvement of occupational therapists in multidisciplinary teams is important, as their expertise in sensory processing and adaptive behaviors addresses factors that contribute to the development and persistence of pediatric feeding disorders. Occupational therapy interventions, such as modifying food textures and enhancing sensory experiences, can play a significant role in promoting more effective feeding behaviors and supporting long-term treatment success) [11].

This scoping review aims to address the following questions: (1) What are the non-pharmacological interventions for managing food selectivity in individuals with ASD? (2) How do sensory, behavioral, and environmental factors influence the outcomes of these interventions? (3) What is the role of occupational therapists in these programs? By answering these questions, this study seeks to provide evidence-based guidance for clinicians and caregivers, ultimately improving care for individuals with ASD.

## 2. Methods

The Preferred Reporting Items for Systematic Reviews and Meta-Analyses Extension for Scoping Reviews (PRISMA-ScR) statement was used for the manuscript reporting [12]. The protocol was registered in the International Prospective Register of Systematic Reviews [PROSPERO: CRD42023476083] and is publicly available.

### 2.1. Eligibility Criteria

Inclusion criteria: studies were selected based on the following criteria: (i) Randomized Controlled Trials, Pilot Randomized Controlled Trials, Pilot Studies, Quasi-experimental Studies, and Case Series; (ii) studies involving participants with a confirmed diagnosis of ASD, with no age or gender differences considered; (iii) studies that report non-pharmacological treatments (psychological, rehabilitative, behavioral, sensory, etc.). Exclusion criteria: data published as abstracts or conference proceedings, observational studies, letters to editors, reviews, meta-analyses, or animal model research.

### 2.2. Information Sources

To identify potentially relevant documents, the following bibliographic databases were searched from 2023 to October 2024: MEDLINE, EBSCO, Web of Science, OTseeker, and SCOPUS. The search strategies were drafted by an experienced librarian (G.G) and further refined through team discussion. The final search strategy for MEDLINE was: ((autism or ASD or autism spectrum disorder or Asperger’s or Asperger syndrome or autistic disorder or Aspergers) AND (food selectivity or eating disorders or anorexia or bulimia or disordered eating or binge eating disorder) AND (treatment or intervention or therapy or management or rehabilitation)), and was subsequently adapted for the other databases. The search was performed to find English-language articles.

### 2.3. Selection of Sources of Evidence

Two independent reviewers (R.S., S.C.) extensively searched MEDLINE, EBSCO, Web of Science, OTseeker, and SCOPUS to identify eligible studies. Each information source was examined from inception to October 2024. References of included papers were reviewed to ensure that no relevant studies were missing through the electronic database searches. We resolved disagreements on study selection and data extraction by consensus and discussion with other reviewers if needed.

### 2.4. Data Charting

A data-charting form was jointly developed by two reviewers to determine which variables to extract. Rayyan QCRI online software was used to remove duplicates and for the study selection [13]. The two reviewers independently charted the data (R.S.; S.C.), discussed the results, and continuously updated the data-charting form in an iterative process. A third reviewer (A.B.) solved disagreements.

#### Data Items

The following data from included studies were extracted by two independent reviewers (R.S., S.C.) using a planned standardized spreadsheet: (i) study design; (ii) aim of the study; (iii) sample size; (iv) mean age (SD); (v) intervention; (vi) intervention duration; (vii) results; (viii) health professionals.

### 2.5. Analysis

Given the heterogeneity of the study designs and outcome measures in the included articles, data synthesis was performed in a structured manner. First, we grouped the studies based on the clinical focus of each reported outcome. Specifically, we categorized them into the following domains: (1) type of intervention and (2) health professionals involved. The data were pooled and analyzed within the most appropriate category for studies reporting multiple outcomes across different domains.

## 3. Results

**Figure 1:**
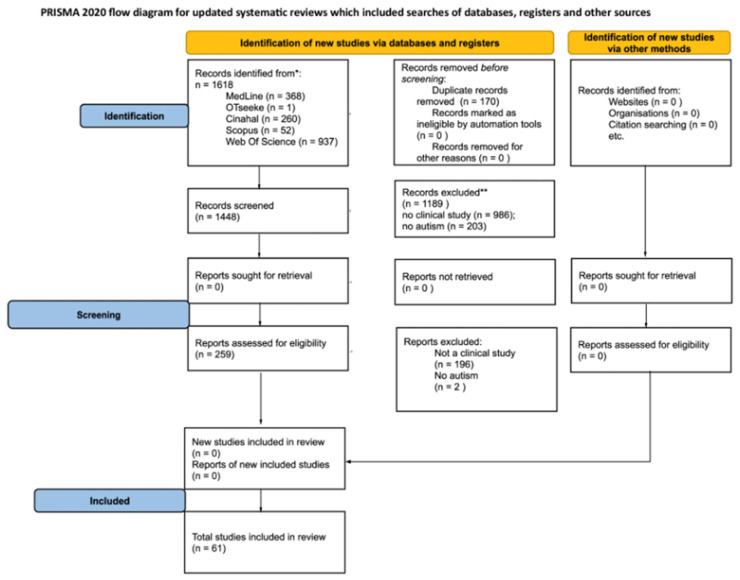
PRISMA Flowchart. * Consider, if feasible to do so, reporting the number of records identified from each database or register searched (rather than the total number across all databases/registers). ** If automation tools were used, indicate how many records were excluded by a human and how many were excluded by automation tools.

As reported in Figure 1, the total number of items retrieved from databases was 1618. Once duplicates were removed (170 searches), the full text of 259 articles was analyzed and 61 articles were included for qualitative synthesis.

As reported in Table 1, sixty-one studies were published between 2001 and 2023. Of these, there were 14 case series [14,15,16,17,18,19]; 12 clinical trials [20,21,22,23,24,25,26,27,28,29,30,31]; 7 pilot studies [32,33,34,35,36,37,38]; 10 between experimental and quasi-experimental studies [39,40,41,42,43,44,45,46,47,48]; 2 retrospective studies [49,50]; 6 prospective studies [51,52,53,54,55,56]; 3 preliminary studies [57,58,59]; 4 qualitative studies [60,61,62,63]; a quantitative follow-up study [64]; and 10 studies between RCT and pilot RCT [5,65,66,67,68,69,70,71,72,73]. All studies analyzed the effects of different non-pharmacological therapeutic approaches aimed at improving feeding difficulties in individuals with ASD. As reported in Table 2, the studies focused on various interventions, including behavioral, sensory, and nutritional approaches. In particular, five studies examined the effects of the “Bringing Adolescent Learners with Autism Nutrition and Culinary Education” (BALANCE) intervention; four evaluated the effectiveness of Applied Behavior Analysis (ABA); four others studied sequential and simultaneous food presentation strategies; three focused on the Managing Eating Aversions and Limited Variety (MEAL) Plan; three examined video modeling techniques; three assessed behavioral interventions based on Differential Reinforcement of Alternative Behavior (DRA) and Escape Extinction (EE); two analyzed the dietary intervention “Easing Anxiety Together with Understanding and Perseverance” (EAT-UP); and two evaluated the Parent Training for Feeding (PT-F) program. Tables were used to report the following main data: author (year), study design, target, sample size, average age of the sample, intervention, duration of intervention, outcome measures, control, professional figures, follow-up, and results. The content and methodology of the studies were qualitatively analyzed, summarizing the main results based on intervention on eating disorders.

### 3.1. Nutritional Intervention “Bringing Adolescent Learners with Autism Nutrition and Culinary Education” (BALANCE)

The BALANCE intervention program incorporates principles of Social and Cognitive Theory (SCT) and techniques for treating food selectivity and feeding difficulties in ASD. This specific intervention was analyzed in five different studies [35,54,60,61,62] and the feasibility of its application in a virtual environment, through the optimization of the theoretical framework, was evaluated in terms of acceptability, benefits, and undesirable consequences. In general, good results were found with regard to: feasibility, attendance rate, completion of tasks, treatment fidelity, and lack of technical difficulties, with improvements in dietary intake levels and self-efficacy. Findings indicate good feasibility, high attendance rates, successful task completion, and treatment fidelity. Positive effects were observed in dietary intake and self-efficacy. However, limitations include small sample sizes and the need for individualized adaptations.

### 3.2. Behavioral Analytical Interventions (ABA)

The ABA method is based mainly on four key principles: reinforcement, extinction, stimulus control, and generalization. Studies using ABA (*n* = 4) consistently reported increased food acceptance and reduced problem behaviors related to feeding. Two of these studies involved an occupational therapist, whose role included supporting sensory adaptations and structuring mealtime environments. However, the effectiveness of ABA may depend on factors such as intervention intensity, individual responsiveness, and co-occurring sensory sensitivities.

### 3.3. Simultaneous and Sequential Presentation Mode of Food

Four studies examined the impact of presenting preferred and non-preferred foods either simultaneously or sequentially. Results were mixed: while sequential presentation, combined with escape extinction, showed promising results in increasing acceptance of non-preferred foods, simultaneous presentation alone was generally less effective. Some studies suggest that combining these methods with reinforcement strategies may enhance their efficacy.

### 3.4. Managing Eating Aversions and Limited Variety (MEAL) Plan

The MEAL Plan is a structured intervention that actively involves parents in addressing food selectivity in children with ASD. Three studies evaluated its effectiveness, reporting improvements in food acceptance and reductions in parental stress. However, successful implementation relies on caregivers’ adherence and consistency, which may present challenges in real-world settings.

### 3.5. Videomodeling

Video modeling is an observational learning technique used to teach appropriate behaviors. Three studies explored its application in home settings, demonstrating positive effects on food-related autonomy and behavior regulation. One study specifically highlighted the role of occupational therapists in structuring and implementing video modeling sessions. However, more research is needed to determine the long-term sustainability of these outcomes.

### 3.6. Behavioral Intervention Based on Differential Reinforcement of Behavior Alternative (DRA) and Leak Quenching (EE)

DRA involves reinforcing appropriate eating behaviors, while EE prevents avoidance behaviors by ensuring continued exposure to non-preferred foods. Three studies examined these techniques, showing increased food variety and consumption. However, the effectiveness of these interventions may be influenced by factors such as individual tolerance levels and parental involvement. In one study, the occupational therapist played a critical role in assessing feeding-related skills and selecting target foods in collaboration with parents [26,57].

### 3.7. Food Intervention Easing Anxiety Together with Understanding and Perseverance (EAT-UP)

The EAT-UP model is a family-centered intervention designed to reduce anxiety and increase food acceptance in children with ASD. Two studies (one follow-up and one preliminary) reported improvements in food acceptance and meal-related behaviors. High procedural fidelity among both clinicians and parents suggests strong feasibility. However, further research is needed to compare EAT-UP with other family-based interventions [59,64].

### 3.8. Parent Training for Feeding (PT-F) Program

The PT-F program trains parents in behavioral strategies to manage mealtime challenges. Two studies found it effective in reducing disruptive behaviors and improving parental confidence. Overall satisfaction with the program was high, but the need for structured follow-up and ongoing support was emphasized [63,65].

From the analysis of the results, several trends emerge: (i) Behavioral interventions (e.g., ABA, DRA/EE, PT-F) show strong effectiveness in increasing food acceptance and reducing mealtime challenges. However, their success depends on consistent implementation and individual responsiveness. (ii) Sensory-based approaches (BALANCE, EAT-UP) demonstrate potential benefits, but further research is needed to directly compare them with behavioral interventions. (iii) Parental involvement is a critical factor in successful outcomes. Programs like MEAL Plan and PT-F highlight the importance of training caregivers, though challenges related to adherence remain. (iv) The role of occupational therapists varies across studies. While some studies integrate OTs into intervention teams, their specific contributions (e.g., sensory adaptations and environmental structuring) are not always clearly defined. Future research should explore their impact in greater detail. (v) Long-term sustainability of intervention outcomes is unclear. Most studies assess short-term effects, and few examine maintenance beyond the intervention period. Longitudinal studies are needed to determine lasting benefits.

This review underscores the importance of integrating multiple approaches to address food selectivity in ASD. Future research should focus on optimizing intervention strategies, defining the role of occupational therapists, and evaluating long-term effectiveness.

## 4. Discussion

This scoping review aimed to address the following questions: (1) What are the non-pharmacological interventions for managing food selectivity in individuals with ASD? (2) How do sensory, behavioral, and environmental factors influence the outcomes of these interventions? (3) What is the role of occupational therapists in these programs? This study aims to offer evidence-based guidance to clinicians and caregivers to enhance care for individuals with ASD. Sixty-one studies were examined, and a critical analysis revealed significant methodological limitations across most of them. Specifically, only 13 studies included a control group [5,16,21,23,30,53,65,66,68,69,70,72,73]; limiting the ability to establish causal relationships between interventions and outcomes. Furthermore, only three studies featured a homogeneous sample in terms of age [22,26,55]; while most studies had small sample sizes (<30 participants), with the exception of eight studies [36,42,49,65,66,70,72,73]. Follow-up assessments were conducted in only 26 studies, indicating that long-term effectiveness remains largely unexplored [15,16,17,18,21,25,26,29,30,32,39,41,47,48,49,51,52,55,56,59,66,67,70,71,72,73]. Several studies were published between 2022 and 2023. Replication studies and larger-scale trials are needed to validate these findings [25,35,43,44,54,58,60,62,67,71,72,73]. Despite these limitations, the analysis of 61 articles highlights the growing involvement of occupational therapists in ASD feeding interventions over time [5,15,20,21,32,33,34,45,49,53,57,70]. Among the included studies, 49 articles focused on sensory and behavioral interventions, 7 on nutritional interventions, 3 on sensory interventions, 1 on muscular reinforcement, and 1 on rehabilitation. However, the lack of standardization in intervention protocols, outcome measures, and participant characteristics makes direct comparisons challenging. Among the studies that concern behavioral interventions, those both statistically and clinically significant are: the study by Marshall et al. (2015) [70], which compared operant conditioning (OC) and systematic desensitization (SysD) in improving food variety and reducing mealtime problem behaviors; Sharp et al. (2014) [69], which evaluated the feasibility and effectiveness of the MEAL Plan study, highlighting its structured approach to managing food selectivity; Laud et al. (2009) [49], which assessed an interdisciplinary feeding program using behavioral treatment strategies; the study by Panerai et al. (2018) [25], which examined a multidisciplinary, intensive behavioral intervention for children with ASD and intellectual disability (ID); the study by Miyajima et al. (2017) [53], which developed and tested a new parenting intervention program to improve selective eating behaviors in children with ASD; and the study by Thorsteinsdottir et al. (2022) [71], investigated the effects of the “Taste Education” program on mealtime behaviors in children with ASD. Among the most studied interventions, the BALANCE program—an 8-week nutritional intervention—has been analyzed in five studies. One study [62] incorporated virtual focus groups, adding an interactive component to the intervention. However, the age range (12–21 years) and the use of varying outcome measures (FFQ, PAS, ABI-SF) limit the generalizability of the findings.

ABA-based interventions lack consistency in the number of weekly sessions, total duration, and assessment methods. Different measurement tools (CEBI, SSIS) were applied across studies, further complicating comparisons. Additionally, participant age ranges varied significantly, reducing the ability to draw generalized conclusions.

In the case of sequential and simultaneous food presentation strategies, only three studies [27,44,63] evaluated their effectiveness, with intervention frequency ranging from two to four sessions per week. These studies lacked standardized outcome measures and included heterogeneous age groups, making it difficult to determine the efficacy of these strategies.

Similarly, MEAL Plan interventions showed heterogeneity in session duration, total treatment length, and participant characteristics. Three studies applied the Brief Autism Mealtime Behavior Inventory (BAMBI), but additional outcome measures varied across studies, limiting comparability.

The video modeling technique was assessed in three studies, but none utilized objective outcome measures. Sample sizes were extremely small (*n* = 3 per study), with significant age variability. Only two studies [15,31] included children of similar ages (3–4 years), making it difficult to determine broader applicability. For DRA and Escape Extinction (EE) interventions, the number and duration of sessions varied significantly among the three studies that examined them. Only one study [41] employed an objective evaluation measure (the Brief Parent Satisfaction Questionnaire), while the other two lacked standardized assessments. Participant age ranges were inconsistent across studies, preventing clear conclusions about the effectiveness of these techniques.

The EAT-UP model, analyzed in two studies, had a treatment duration of 5–6 months. Effectiveness was assessed using BAMBI and FFQ in both studies, with one study [59] additionally employing the Behavioral Pediatrics Feeding Assessment Scale (BPFAS). However, age heterogeneity across studies limits generalizability.

Finally, the Parent Training for Feeding (PT-F) program lacks sufficient data to determine the optimal age group, session frequency, and duration of effectiveness. The two studies that examined this intervention used multiple evaluation tools (BAMBI, PSQ, Treatment Fidelity Checklist, ABC, PSI-Short Form), making cross-study comparisons difficult.

This review highlights key clinical implications. Clinicians should implement structured behavioral interventions, such as ABA-based programs, with standardized protocols and assessments. Moreover, sensory-based interventions like BALANCE and EAT-UP should be further explored in larger studies to confirm their efficacy. A key recommendation for clinicians is to actively involve caregivers in intervention plans, as programs like MEAL and PT-F show that parental participation greatly enhances treatment adherence and success. Lastly, clinicians should advocate for long-term follow-up assessments to better understand the sustainability of intervention effects over time.

The findings of this review have important implications for clinical practice:

(i) Behavioral interventions, particularly ABA-based approaches, show promise but require greater standardization in treatment protocols, session duration, and outcome assessments. (ii) Sensory-based interventions, such as BALANCE and EAT-UP, offer potential benefits, but their effectiveness needs to be validated through larger, well-controlled studies. (iii) Parental involvement plays a crucial role in intervention success. Programs like MEAL and PT-F highlight the importance of training caregivers, yet adherence and implementation fidelity remain challenges. (iv) The role of occupational therapists in feeding interventions is increasing, but future research should focus on quantifying their specific contributions to treatment outcomes. (v) There is a lack of long-term follow-up studies, making it difficult to assess the sustainability of intervention effects over time.

The role of occupational therapists in feeding interventions is increasingly recognized, but their specific contributions need further clarification. Occupational therapists apply a holistic, occupation-based approach that focuses on feeding as an essential daily activity within various life contexts. Their interventions include sensory integration techniques, adaptive strategies for mealtime participation, and caregiver training to enhance feeding routines. Several studies [5,15,20,21,32,33,34,45,49,53,57,70] highlight their involvement in feeding programs, demonstrating their role in improving sensory processing, motor coordination, and behavioral adaptations during mealtimes. Future research should aim to quantify the impact of occupational therapy interventions within multidisciplinary teams, ensuring their systematic integration into feeding programs for individuals with ASD. To address the variability in methodologies across studies, future research should prioritize the standardization of intervention protocols and outcome measures. Establishing uniform assessment criteria, such as using validated tools like BAMBI and FFQ across studies, would facilitate comparability and improve the reliability of findings. Furthermore, defining optimal session duration, frequency, and intervention length for ABA-based and sensory interventions would enhance the applicability of results in clinical practice. Standardized methodologies would also support the development of meta-analyses, enabling a more comprehensive understanding of effective strategies for managing food selectivity in ASD.

Future research should prioritize larger, well-designed RCTs to establish causal relationships between interventions and outcomes. Standardizing outcome measures will improve cross-study comparability and support meta-analyses. Additionally, long-term follow-ups are needed to assess intervention sustainability. Finally, further research is needed to clearly define the specific role of occupational therapists within interdisciplinary feeding programs, ensuring their contributions are well understood and effectively integrated into treatment approaches. This review highlights the need for more rigorous research methodologies to strengthen the evidence supporting non-pharmacological interventions for food selectivity in ASD. Addressing these gaps will enhance the practical application of interventions and improve outcomes for individuals with ASD and their families.

## 5. Conclusions

This review highlights the variety of non-pharmacological interventions for food selectivity in ASD, though their effectiveness is limited by methodological inconsistencies. Stronger evidence is needed through larger, well-designed RCTs with standardized outcome measures and longer follow-up periods. A key finding is the growing role of occupational therapists (OTs) in feeding interventions. OTs address sensory sensitivities and behavioral challenges, yet their specific impact remains underexplored. Future research should clarify their role and effectiveness within interdisciplinary teams. Clinically, integrating behavioral, sensory, and nutritional strategies appears most effective. Standardized protocols are needed to improve implementation and outcomes. Advancing research in these areas will provide clearer guidance for practitioners and caregivers.

## Figures and Tables

**Table 1:** Data extraction table.

Author (Year)	Study Design	Aim of the Study	Sample Size	Mean Age (Sd-Range)	Outcome Measures	Follow-Up	Health. Professionals
Johnson et al. (2019) [65]	Clinical trial	Evaluate the effectiveness of a parent training program (PT-F) aimed at integrating behavioral strategies and nutritional guidance.	42	5.1	BAMBI-revised, Stanford–Binet Intelligence test, Mullen Scales of Early Learning, ps, treatment fidelity checklist, about your child’s eating (ayce), clinical global impression–improvement scale (cgi-i), aberrant behavior checklist (abc), home situations questionnaire (hsq), parenting stress index (psi), the parenting sense of competence (psoc) scale, caregiver strain questionnaire (cgsq)	N/A	Dietitian, therapist, and psychologist
Sharp et al. (2019) [66]	Clinical trial	Assess the effectiveness of the meal plan (managing eating aversions and limited variety) in children with autism spectrum disorder (ASD) and moderate food selectivity.	38	58.7 (±13.8; 38–88 months)	Clinical global impression–improvement scale, BAMBI	20th week	Psychologist and dietitian
Peterson et al. (2016) [5]	Randomized pilot study	Compare the m-sos approach with the ABA approach for treating food selectivity.	6	N/A	N/A	N/A	Certified occupational therapist ABA and m-sos
Crowley et al. (2020) [14]	Case series	Evaluate the effects of an intervention based on the matching law on consuming an age-appropriate, nutritionally adequate diet.	7	N/A	N/A	N/A	Psychologist
Burrell et al. (2023) [67]	Randomized pilot study	Assess the impact of child, parent, and feeding behavior characteristics on response to treatment based on the meal plan.	19	N/A	Social communication questionnaire (scq)—lifetime, demographic form, ADOS, Stanford–Binet Intelligence Scales—fifth edition, differential ability scales—second edition (das-ii), Mullen Scales of Early Learning, parent/caregiver form of the vineland ii, BAMBI, clinical global impression–improvement scale (cgi-i), aberrant behavior checklist (abc)	1/2 months	Psychologist and dietitian
Hillman (2019) [15]	Case series	Evaluate the effects of video modeling in a home setting on food selectivity	3	4.7 (3–4)	N/A	5 months	Occupational therapist
Taylor et al. (2017) [20]	Clinical trial	Compare the effectiveness of applied behavior analytic interventions in addressing feeding difficulties and tube dependency in children with ASD and cerebral palsy.	25	69.53 (±30.69; 20–148) months	Children’s eating behavior inventory (cebi)	N/A	Pediatrician, gastroenterologist or nurse, behavioral therapist or psychologist, dietitian, and oral motor therapist (occupational therapist or speech therapist)
Peterson et al. (2019) [68]	Randomized controlled trial	Assess the effects of an ABA-based intervention on independent acceptance and mouth clearing of healthy, novel, and non-preferred foods	6	3.3 (±0.82)	N/A	N/A	ABA therapist
Kuschner et al. (2017) [32]	Pilot study	Evaluate the efficacy of the buffet cognitive-behavioral treatment for food selectivity in children with ASD	11	N/A	Social communication questionnaire, differential abilities scales-ii, client satisfaction questionnaire (csq-8),	4 and 12 weeks	Doctor, nurse, occupational therapist, nutritionist, psychologist, dietitian, social worker
Seiverling et al. (2018) [21]	Case series	Compare a behavioral feeding intervention with and without pre-meal sensory integration therapy (sit).	2	6.5 (5.2–6.1)	Child sensory profile-2 (csp-2),	2 months	SLT, OTR/L, dietitian, feeding therapist (certified behavior analyst), registered behavioral technician, psychologist
Turner et al. (2020) [22]	Case series	Examine the effects of a response-shaping procedure using a wide set of rotating foods and a small set of consistent foods on food acceptance.	2	6 (±0)	N/A	N/A	N/A
Levin et al. (2001) [23]	Case series	Evaluate the effects of the presence or absence of positive reinforcement on the effectiveness of feeding interventions for children with ASD and food selectivity.	3	N/A (5–7)	Stanford–Binet Intelligence Scales	N/A	Special education teachers, nutritionists, experimenters, university research assistants (primary observers), and authors
Taylor (2020) [39]	Case series	Demonstrate the effectiveness of non-removal of the spoon and physical guidance in increasing food consumption and reducing inappropriate mealtime behaviors.	2	N/A (4–5)	N/A	1.5–3 years	Psychologists, MD
Sharp et al. (2014) [69]	Pilot randomized controlled trial	Describe and assess the feasibility and content of the autism meal plan program and study protocol.	19	Experimental: 70.8 (±20.5; 36–104); control: 64.8 (±16.9; 45–94) (months)	Social responsiveness scale (srs), food preference inventory (fpi), BAMBI, the parenting stress index-short form (psi-sf), social validity, and parent perception of improvement.	N/A	Pediatric psychologist
Silbaugh et al. (2019) [24]	Case series	Evaluate the generalizability of the high-probability sequence (hps) by applying it to feeding interventions.	3	5 (±1; 4–6)	Feeding questionnaire stimulus preference assessments (spas), hierarchical feeding compliance assessments (hfca), reinforcer assessments (ra), general questionnaire administration, mealtime journal, and semistructured mealtime observation.	N/A	N/A
Marshall et al. (2015) [70]	Randomized controlled trial	Determine whether operant conditioning (oc) or systematic desensitization (sysd) leads to improvements in food variety and reductions in problematic mealtime behaviors.	68	Experimental: 50.1 (±12.4); control: 50.6 (±10.5) (months)	Parent evaluation of developmental milestones—assessment version (peds-dm), Behavioral Pediatrics Feeding Assessment Scale (BPFAS), eyberg child behavior inventory (ecbi), parenting stress index-short form (psi-sf)	3 months	“pediatrician, psychiatrist, speech therapist, occupational therapist, psychologist, and nutritionist”
Laud et al. (2009) [49]	Clinical trial	Evaluate the outcomes of a behavioral treatment program within an interdisciplinary feeding program.	46	69 (36–145 months)	Children’s eating behavior inventory (cebi), caregiver satisfaction scores	40 months	Gastroenterologist, pediatrician, nurse, nutritionist, occupational therapist and/or speech therapist, and social worker
Fu et al. (2015) [51]	Case series	Assess the effectiveness of contingency affirmation and modeling in increasing food consumption in children with food selectivity.	2	N/A (9–10)	N/A	4 and 8 weeks	Feeding therapists, ABA interns, and treatment authors
Panerai et al. (2018) [25]	Clinical trial	Present the results of a multidisciplinary, intensive, day-treatment behavioral feeding program for children with ASD and intellectual disabilities (id).	8	N/A	BAMBI-18, psychoeducational profile third edition (pep-3), autism diagnostic interview-revised o ados o autism rating scale-second edition	1 year	Multidisciplinary team, ABA-qualified therapist, speech therapist, nutritionist, psychologist or psychotherapist, and feeding therapists
Ulloa et al. (2020) [57]	Pilot study	Evaluate the effects of omission and commission errors on the effectiveness of differential reinforcement of alternative behavior (dra) intervention with escape extinction.	3	N/A (3–5)	N/A	N/A	“interdisciplinary team composed of behavior analysts, pediatrician, nurse, nutritionist, occupational therapists, speech therapists, and social worker.”
Paul et al. (2007) [52]	Case series	Evaluate the effects of repeated food exposure on increasing food variety.	2	N/A (3.5–5)	N/A	3 months	N/A
Vandalen and Penrod (2010) [47]	Case series	Compare sequential and simultaneous presentation methods without escape extinction for treating food selectivity, and assess generalization and maintenance of food consumption.	2	N/A (4–5)	N/A	2–4–6–12 weeks	N/A
Penrod and VanDalen (2010) [27]	Case series	Conduct a sequential analysis of intervention components: bite presentation, reinforcement importance, and escape prevention.	3	N/A (3–4)	Satisfaction questionnaire for the parents	2–4–6–12 weeks	Therapist
Russo et al. (2019) [26]	Case series	Assess the effectiveness of a treatment package to increase food consumption and decrease (or maintain low) latency to consumption using differential reinforcement of alternative behavior, choice as an antecedent strategy, and escape extinction.	2	17	N/A	12 weeks	N/A
Penrod and Vandalen (2010) [27]	Case series	Evaluate whether sequential and simultaneous exposure procedures increase preference for non-preferred foods (npfs).	3	N/A (4–5)	N/A	N/A	N/A
Burton et al. (2021) [28]	Case series	Demonstrate the use of the fbt + up approach to treat arfid in children with ASD in a clinical setting.	2	N/A (6.1–11.5)	Up-c parent modules	N/A	Dietitian, psychologist, speech therapist, medical team
Miyajima et al. (2017) [53]	Clinical trial	Develop and evaluate a new parent intervention program aimed at improving selective eating behaviors in children with ASD.	23	N/A (3–6)	A visual analog scale (vas), self-efficacy assessment for parents of children with selective eating (saps) scale	N/A	Occupational therapist
Harpster et al. (2015) [33]	Pilot study	Apply the engagement–communication–exploration (ece) intervention during snack time to promote food exploration, consumption, and social engagement in young children with ASD.	6	N/A (3–4)	Frequency questionnaire (FFQ), BAMBI	N/A	A team of occupational therapists, kindergarten teachers, graduate students, and independent observers
Buro et al. (2022) [54]	Clinical trial	Examine the feasibility of implementing the virtual nutritional intervention bringing adolescent learners with autism nutrition and culinary education (balance).	27	15 (12–20)	Food frequency questionnaire (FFQ), physical activity screener (pas), demographic questionnaire and the autism behavior inventory—short form (abi-s), block kids FFQ, psychosocial survey and FFQ + pas	N/A	Nutritionist
Gale et al. (2011) [29]	Case series	Analyze the mealtime behavior of three children with ASD to develop individualized interventions based on functional assessment results.	3	N/A (30–52)	Icd-10, Autism diagnostic interview—revised (adi-r)	4/5 months	Family doctor, ABA tutor, first author, and second observer
Flanagan et al. (2021) [30]	Case series	Evaluate the effectiveness of contingency modeling in increasing food consumption in three children with ASD and food selectivity.	3	9.1 (6–10)	Communication domain of the vineland adaptive behavior skills—3rd edition	N/A	Experimenter
Seiverling et al. (2020) [50]	Clinical trial	Examine changes in children’s mealtime behavior, diet variety, and family dynamics after an intensive interdisciplinary behavioral treatment (iibt) for feeding problems.	16	52.56 months	About your child’s eating scale (ayce), BAMBI	N/A	Interdisciplinary team, including a speech therapist, dietitian, psychologist, pediatric nurse, pediatric gastroenterologist, and behavioral therapists
Buro et al. (2022) [60]	Clinical trial	Optimize a theoretical framework based on social cognitive theory (sct) for the virtual balance nutritional education intervention.	27	N/A (12–21)	N/A	N/A	Nutritionist
Buro et al. (2021) [61]	Clinical trial	Examine the acceptability, perceived benefits, and unintended consequences of the virtual implementation of the balance program.	27	N/A (12–21)	N/A	N/A	Nutritionist
Cihon et al. (2021) [40]	Clinical trial	Evaluate the impact of the virtual balance food education program on healthy food consumption in adolescents and young adults with ASD.	3	N/A (5.5–11)	Social skills improvement system (ssis)	N/A	First author (educator and behavior analyst) and research assistant
Suarez and Bush (2020) [34]	Case series	Assess the feasibility and preliminary efficacy of the Autism Eats program to improve food intake and mealtime behaviors in children with ASD.	4	N/A (2–13)	Short sensory profile 2 (ssp2)	N/A	Occupational therapist and first author
Najdowski et al. (2010) [41]	Case series	Evaluate the adaptation and implementation of a nutritional program for children with ASD and their parents.	3	N/A (2–4)	A brief version of a psq developed by Hoch, Babbitt, Coe, Krell, and Hackbert (1994).	2, 4, 6, and 12 weeks	Primary researchers, 2 independent observers, therapists, and researchers
Thorsteinsdottir et al. (2022) [71]	Randomized contolled trial	Analyze the effects of oral motor work in children with ASD and food selectivity and assess whether an additional home-based program by an occupational therapist reduces parental concerns.	7	N/A (8–12)	Questionario meals in our household-mioh	6 months	Psychologist, nutritionist, 4 taste educator assistants
Chung et al. (2020) [42]	Clinical trial	Develop and evaluate a group intervention for individuals with ASD to increase healthy food consumption, particularly examining pre-meal presentation effects on vegetable consumption in a school setting.	56	10.7 (±2.2; 8–15)	Diagnostic and statistical manual of mental disorders: 5th edition, BAMBI, habitual fv consumption	N/A	Research staff
Zhou et al. (2023) [43]	Case series	Examine whether improvements from a behavioral intervention are maintained when parents continue the program at home and during meals outside the home.	4	N/A (4.8–10.5)	N/A	N/A	A total of 4 primary research assistants (2 special education teachers and 2 certified behavior analysts—bcba), doctoral-level bcba researchers (bcba-d)
Davis et al. (2023) [44]	Case series	Provide written instructions for video modeling, along with tips and feedback, to train parents in applying structured meal procedures.	3	N/A (6–13)	N/A	N/A	Bcba therapists, school staff trained in ABA techniques and cpr, and nurses
Kral et al. (2023) [72]	Clinical trial	Evaluate the effectiveness of video modeling of contingencies combined with direct contingency exposure in treating food selectivity.	38	Experimental: 8.9 ± 1.2; control: 8.4 ± 1.4	Digital scale (seca 876), portable stadiometer (seca 217), short-form sensory profile	3 months	Research staff
Buro et al. (2023) [62]	Clinical trial	Test the initial effectiveness, feasibility, and acceptability of a parent-based weight management intervention for children with ASD (pbt-ASD).	27	15 (12–19)	N/A	N/A	Nutritionist
Buro et al. (2022) [35]	Pilot study	Investigate the effects of a preventive program using repeated vegetable exposure on preschool children with ASD without severe food selectivity.	27	N/A	Block kids 2004 food frequency questionnaire (FFQ), physical activity screener (pas), sct-based survey developed and evaluated, online survey platform-qualtrics, the block kids pas (nutrition-quest), a digital bathroom scale (letsfit eb5636 h, letsfit), e autism behavior inventory—short form	N/A	Nutritionist
Gray et al. (2022) [73]	Pilot randomized controlled trial (RCT)	Conduct a follow-up study on the eat-up pilot intervention previously completed by the same authors.	48	N/A	BAMBI, cfq, portable stadiometer (seca 213), digital weight scale (tanita wb-800 as), nhanes anthropometry procedures manual	5 months	Staff and research assistants
Manzanarez et al. (2021) [36]	Pilot study	Evaluate the efficacy of a family-centered feeding intervention, easing anxiety together with understanding and perseverance (eat-up), in promoting food acceptance in children with ASD in home settings.	50	N/A	N/A	N/A	Cadd psychologists, knf coordinator, program coordinator, 1 promoter, 1 ABA therapist, and 3–4 volunteers
Khan et al. (2021) [45]	Clinical trial	Conduct a parent training program for children with ASD focusing on integrating behavioral strategies and nutritional guidance (PT-F) to assess feasibility and initial effectiveness.	15	N/A	The Com deall Oro Motor Assessment Scale, Behavioral Pediatric Feeding Assessment scale	N/A	Occupational therapist
Ivy et al. (2022) [58]	Pilot study	Evaluate the effectiveness of different combinations of redistribution, swallowing facilitation, and packaging reduction in two children with ASD.	16	13	Healthy eating survey, step-child	N/A	A total of 41 school collaborators, researchers, behavior analysts, second independent observers
Taylor et al. (2021) [16]	Case series	Assess the effects of an antecedent-based intervention on increasing food consumption in two children with ASD and food selectivity.	20	6		2.3 years	Therapist (first author), qualified assistant, doctor
Clark et al. (2020) [31]	Case series	Evaluate the effects of individualized reinforcement and hierarchical exposure on increasing food flexibility in three children with ASD with rigid and restrictive mealtime behaviors.	3	N/A (3–6)	N/A	N/A	Experimenter, observer
O’Connor et al. (2020) [19]	Case series	Analyze a treatment package based on repeated taste exposure, escape extinction, and fading demands to increase acceptance of various foods in three children with ASD and food selectivity.	3	N/A (5–12)	N/A	N/A	Research staff
Matheson et al. (2019) [37]	Pilot study	Test the effectiveness of sequential and simultaneous exposure procedures on increasing preference for non-preferred foods (npfs).	20	9.90 (±2.31; 6–13)	Block food frequency questionnaire, finish leisure time physical activity questionnaire	N/A	Clinical psychologist (kb)
Kim et al. (2018) [46]	Clinical trial	Present the application of the fbt + up approach for treating arfid in children with ASD in clinical settings.	27	4.42 ± 0.50 (experimental); 4.04 ± 1.02 (control)	N/A	N/A	Therapists, assistants, and first author
Muldoon and Cosbey (2018) [64]	Case series	Investigate the effects of a taste education program on reducing problematic mealtime behaviors in children with ASD.	3	N/A (3–5)	BAMBI, BPFAS	N/A	Behavioral technicians (rbt), first author, behavior analyst, and speech therapist
Cosbey and Muldoon (2017) [59]	Case series	Assess the effects of the physical transformation of fruits and vegetables into snacks to improve sensory approval in children with ASD.	3	N/A (6–8)	BAMBI, FFQ, BPFAS	6 months	Researchers with expertise in speech therapy and behavioral analysis (ABA approach)
Johnson et al. (2015) [38]	Pilot study	Evaluate the feasibility and effectiveness of a self-control training program to reduce food-stealing behaviors.	14	4 (N/A)	BAMBI, ados, cognitive assessment, psq, treatment fidelity checklist, aberrant behavior checklist (abc), parenting stress index (psi)-short form, 3-day food records (3 dfrs)	N/A	Certified ABA-qualified therapist
Levin et al. (2014) [55]	Case series	Compare two food presentation methods (simultaneous and sequential) in an alternating treatment design to determine effects on target food consumption in three children with ASD in a school setting.	2	4 (N/A)	N/A	2, 4, and 6	Aba therapists, psychologists, behavior analysts, observers
Penrod et al. (2012) [56]	Case series	Test the initial effectiveness of a healthy nutritional intervention on improving the consumption of healthy foods/beverages (fruits, vegetables) and reducing unhealthy options (sweets, sugary drinks) in children with ASD.	2	N/A (9–10)	N/A	3, 6, and 12 weeks	Researchers and independent observers
Koegel et al. (2012) [17]	Case series	Examine the acceptability, perceived benefits, and unintended consequences of implementing the balanced nutritional intervention in a virtual environment for adolescents with ASD.	3	N/A (6.4–7.8)	N/A	N/A	Psychologist, supervised by a psychiatrist
Seiverling et al. (2012) [18]	Case series	Investigate the impact of the balance virtual food education program on healthy food intake in adolescents and young adults with ASD.	3	N/A (4–8)	N/A	Weekly	Researchers

**Table 2:** Intervention type.

Author (Year)	Intervention Type	Intervention Durations	Results
Johnson et al. (2019) [65]	Pt-f	20 weeks; 11 sessions of 60–90 min	This study provides evidence of feasibility, satisfaction, and fidelity in implementing PT-F for feeding problems in young children with ASD.
Sharp et al. (2019) [66]	Meal	16 weeks; 10 base sessions and 3 additional sessions (N/A)	For the meal plan, dropout rates were <10% and participation was >80%. Therapists achieved fidelity > 90%. On the cgi-improvement scale, positive response rates were 47.4% for the meal plan and 5.3% for parent education (*p* < 0.05).
Peterson et al. (2016) [5]	ABA	N/A; 3 sessions/week. Maximum 12 sessions for m-sos and 19 for ABA of 1.5 h	Target food consumption improved for children undergoing ABA treatment but not for those following the m-sos program.
Crowley et al. (2020) [14]	Others	N/A; 2–5 sessions/week, meals from 1 to 4/day (N/A)	When given a choice between a resistant-to-change food and an alternative food (free-choice condition), participants predominantly chose the resistant-to-change food, with few exceptions. Consumption of resistant foods occurred almost exclusively in the free-choice condition due to a long history of producing multiple sources of automatic reinforcement, while no programmed reinforcement was available for consuming alternative foods.
Burrell et al. (2023) [67]	Meal	12 weeks; 10 sessions of 90 min	Higher maternal education and greater baseline communication skills in the child were associated with a positive treatment response. Improvements in table manners and reductions in disruptive mealtime behaviors contributed to treatment success. Results also suggest that the individually delivered meal plan offers greater flexibility compared to group-based intervention for some parents.
Hillman (2019) [15]	Video modeling	N/A; 4–6 and 8 individual evaluation sessions,	No statistical significance was observed for the results of the evaluation scales.
Taylor et al. (2017) [20]	ABA	10 years; (N/A); (N/A)	Video modeling alone led to increased food acceptance among participants; when reinforcement was added, food acceptance levels increased for all three participants in terms of the number of bites.
Peterson et al. (2019) [68]	ABA	N/A; 1 session/week (increased to 3 per week for 2 children) of 1.5 h	The behavioral treatment components were similar between groups and primarily consisted of escape extinction (e.g., non-removal of the spoon) and differential reinforcement. For both groups, behavioral treatment was equally effective in increasing gram consumption and reducing refusals and negative vocalizations. Both groups achieved a high percentage of individualized goals and reported high caregiver satisfaction.
Kuschner et al. (2017) [32]	Buffet	16 weeks; 1 session/week of 90 min	The percentage of independent acceptance and mouth clearing increased for the ABA intervention group, with an addition of 16 foods consumed, but not for the waitlist control group until the intervention was implemented.
Seiverling et al. (2018) [21]	Sensory integration	N/A; 4 sessions/day of 20 min, with 45 min rest between sessions	All parents of children who participated in the buffet program reported being very satisfied; however, only half indicated that their therapeutic needs were fully addressed. Data analysis also revealed that children preferred hands-on and individualized exposure activities over psychoeducational activities.
Turner et al. (2020) [22]	Response Modeling	N/A; up to 79 sessions, 2 sessions/day (N/A)	For both participants, food and beverage consumption and total intake increased to similar levels, with a decrease in inappropriate mealtime behaviors (imb) in both conditions. The sit condition was subsequently discontinued, but both participants continued to exhibit high levels of consumption and low levels of imb during a non-sit phase. Caregivers of both participants were then trained in the behavioral feeding intervention. Follow-up data were collected for only one participant.
Levin et al. (2001) [23]	Others	N/A; at least 11 total sessions (N/A)	For one participant, the two procedures were similar in terms of efficiency, although, in the broad-set condition, many more foods were consumed. For the other participant, the training succeeded in increasing some acceptance behaviors (e.g., placing food in the mouth) but not in the consumption of a large variety of new foods.
Taylor (2020) [39]	Others	15–21 days; (N/A); about 8 h	Participants exhibited significantly more problematic behaviors during the non-preferred food condition. Target non-preferred foods were consumed only after access to preferred foods, when food was restricted, and when a positive reinforcement contingency was implemented. The functional analysis also suggested that problem behaviors were maintained by negative reinforcement.
Sharp et al. (2014) [69]	Meal	N/A; 8 group sessions of 1 h	Admission goals were achieved. For both participants, a >98% reduction in latency to acceptance, a 100% reduction in inappropriate mealtime behaviors, and a 100% increase in food consumption were observed. Food variety increased to over 85 foods with regular textures. Caregivers were trained in the procedure’s implementation, and the protocol was generalized to schools and communities. Results were maintained at 3 and 1.5 years.
Silbaugh et al. (2019) [24]	Hps	N/A; 1–3 sessions/day, 2–3 days/week (N/A)	The hps (high-probability teaching sequence) did not improve the feeding responses of children undergoing the treatment program.
Marshall et al. (2015) [70]	Operational conditioning	10 weeks or 1 week; 10 sessions over 10 weeks, or intensive mode in 1 week (N/A)	No statistically significant differences were found in the outcomes between the oc and sysd intervention groups from baseline to the 3-month follow-up. Clinically significant differences were greater for the oc group.
Laud et al. (2009) [49]	N/A	47 days; about 10 sessions (N/A)	A retrospective graphical analysis indicated that these children were successfully treated, and follow-up data suggest that improvements were maintained even after the program ended.
Fu et al. (2015) [51]	Contingent modeling	N/A; 3–4 sessions, twice a week (N/A)	Consumption modeling alone was insufficient to increase food intake and decrease inappropriate mealtime behaviors. These results suggest that asserting and modeling the consequences of a behavior, rather than just modeling the behavior itself, is more likely to lead to imitation, i.e., consumption. Dr modeling for consumption was not as effective but succeeded in increasing the consumption of two out of three foods for one participant. Dr and nrs models were found to be more effective at increasing food intake compared to dr modeling alone.
Panerai et al. (2018) [25]	N/A	10 weeks; 3 sessions/day of 10–15 or 30 min	Statistically significant differences were found between pre-treatment, post-treatment, and follow-up, with a decrease in problem behaviors during treatment, an increase in body weight, an improvement in chewing effectiveness, and an increase in food variety (both type and texture). Non-parametric statistics were used. Intragroup comparisons were made using the Wilcoxon test, and statistical significance was set at *p* < 0.05. Intergroup comparisons were made using the Mann–Whitney u test, with statistical significance set at *p* < 0.05. Effect sizes were calculated using Cliff’s delta. Finally, for frequency data, the chi-square test was used (*p* < 0.01).
Ulloa et al. (2020) [57]	DRA and EE	N/A; 10–12 sessions/day, 5 sessions/week of 5 m33 s	For one participant, integrity errors became detrimental to treatment when integrity levels were reduced to 40%. For the other two participants, tangible contingencies, attention, and escape extinction remained effective despite being implemented with low integrity. This preliminary demonstration suggests that behavioral interventions for pediatric feeding refusal remain effective despite significant degradation in treatment integrity.
Paul et al. (2007) [52]	N/A	13–15 days; (N/A); (N/A)	This study demonstrated the effectiveness of the combined intervention of repeated taste exposure and escape prevention for treating severe food selectivity. For the tasting sessions, data were collected based on the time spent before consuming the food portion and the number of foods that met the criterion of a full spoon (up to 89% for one participant, and between 72 and 100% for the other, during generalization sessions), as well as the number of sessions needed to meet this criterion. Data were also recorded based on the frequency of inappropriate behaviors during each session and calculated as the percentage of occurrence per total number of trials (decreased from 68% to 14%).
Vandalen and Penrod (2010) [47]	Simultaneous and sequential presentation	N/A; 2 assessment sessions and 1 session/day, 2–3 days/week of 30–45 min	Although the results for two participants indicated that both methods of presentation led to increased levels of acceptance and consumption of non-preferred foods, when combined with an escape extinction procedure, observations suggested that participants preferred consuming foods when presented sequentially.
Penrod and VanDalen (2010) [27]	N/A	N/A; 1 session/day every day of the week of 30 min	It has been shown that treatment packages that include differential reinforcement of alternative behavior (dra) and escape prevention, in the form of a non-removal of the spoon procedure, result in increased food consumption. However, when these treatment components are introduced simultaneously, it is not possible to determine which component(s) are responsible for the behavioral change. For two participants, food consumption did not increase until escape prevention was introduced. For one participant, food consumption increased after the reinforcement magnitude was increased (escape extinction was unnecessary). Results were maintained at a 12-week follow-up for all participants.
Russo et al. (2019) [26]	DRA and EE	N/A; (N/A); (N/A)	The results demonstrated that the treatment package consisting of antecedent choice, differential reinforcement of alternative behavior, and escape extinction increased both food consumption and the variety of foods consumed for each of the participants. Additionally, the use of a changing-criterion design provided a systematic method to increase bite consumption per meal for each participant.
Penrod and Vandalen (2010) [27]	Simultaneous and sequential presentation	N/A; 2–3 sessions/week of 30 min	Neither of the two presentation methods (simultaneous and sequential) proved effective in increasing food consumption; consequently, both presentation methods were combined with escape prevention. After the introduction of this procedure, food consumption increased in both conditions.
Burton et al. (2021) [28]	N/A	Case 1: 8 months; Case 2: 7 months; 24–29 sessions of 15–50 min	It has been shown that treatment packages, including differential reinforcement of alternative behavior (dra) and escape prevention, in the form of a non-removal of the spoon procedure, lead to increased food consumption. However, when these treatment components are introduced simultaneously, it is not possible to determine which component(s) are responsible for the behavioral change. For two participants, food consumption did not increase until escape prevention was introduced. For one participant, food consumption increased after the magnitude of reinforcement was increased (escape extinction was unnecessary). Results were maintained at a 12-week follow-up for all participants.
Miyajima et al. (2017) [53]	N/A	2 months; 2 sessions of 40 min	Significant differences were observed in the level of difficulty perceived by parents, their sense of self-efficacy, the number of recommendations they provided, their view of the degree of food imbalance, and the number of food products consumed by their children, before and after the intervention.
Harpster et al. (2015) [33]	Ece	5 weeks; (N/A); (N/A)	In this pilot study, children improved their social initiation rates (3/6), social responses (2/6), and food exploration (4/6). No significant changes were observed in the FFG or bambic from baseline to post-intervention. Therefore, half of the children benefited from the treatment, particularly in terms of social competence.
Buro et al. (2022) [54]	Balance	8 weeks; 1 session/week of 45 min	The virtual implementation of the balance program was evaluated as feasible, with 88% attendance, high participation (3.5 out of 4), 52% completion of tasks, 99% fidelity, and no major technical difficulties. A total of 93% of participants completed all eight sessions. However, field data indicated that some adolescents were distracted by other devices during treatment sessions. This program aimed to engage adolescents with autism in nutrition and culinary education.
Gale et al. (2011) [29]	Others	N/A; 51 total sessions, 2 sessions/day baseline, 5 sessions/day treatment of 10 min	In the current study, the fao provided more detailed information than the fai. However, the fai proved to be more essential for identifying feeding problems. Therefore, both the fai and fao were necessary to identify the goal, intervention methods, and the most appropriate objectives.
Flanagan et al. (2021) [30]	Contingent modeling	N/A; 3–4 sessions/day of 5 min	Participants initially showed positive results when reinforcement was contingent on consuming all target foods on the plate. However, during treatment sessions, this contingency became ineffective for two participants, leading to the introduction of the spoon non-removal modeling technique, which increased food consumption for both participants.
Seiverling et al. (2020) [50]	Iibt	2–8 weeks; 4 sessions/day, mon-fri of 45 min	All outcomes of this feeding program, except for fruit acceptance, showed significant improvements from pre- to post-intervention. Current results suggest that iibt is effective in improving feeding behaviors in many children, regardless of their developmental or medical status.
Buro et al. (2022) [60]	Balance	8 weeks; 1 session/week of 45 min	The results suggest that future versions of balance intervention should incorporate sdt (self-determination theory, based on three constructs: self-regulation, autonomy and support, and the social environment) to enhance the intrinsic motivation of adolescents to make healthy food choices. The optimized framework could inform future virtual nutrition education programs for this population.
Buro et al. (2021) [61]	Balance	8 weeks; 1 session/week of 45 min	The results suggest that the virtual implementation of balance was acceptable to adolescents with ASD and their parents. Based on participant feedback, many adolescents with ASD could benefit from small-group interventions, and virtual interventions offer a convenient option for some adolescents and parents.
Cihon et al. (2021) [40]	Aba	N/A; 1 session/day for max 5 days/week of 10 min	All three participants began selecting ilp foods (initially low preference) only after the intervention was introduced. However, the results varied among the three participants.
Suarez and Bush (2020) [34]	Just right challenge	12 weeks; 1 session/week of 60 min	After a latency period following treatment, five out of seven children accepted a significantly larger amount of food. These five children each had sensory profiles indicating possible sensory hyperreactivity. There was a statistically significant increase from baseline to the last treatment session in the average number of foods in the group’s inventory. The just right challenge feeding protocol is, therefore, a promising treatment for increasing food acceptance in some children with food selectivity and sensory hyperreactivity (*p* = 0.018).
Najdowski et al. (2010) [41]	Dra e ee	N/A; 2–7 sessions/week of up to 30 min	This study demonstrated that mothers trained to implement dra (differential reinforcement of alternative behavior) combined with nrs (non-removal of the spoon) and demand reduction could be effective in increasing the consumption of non-preferred foods by their children and decreasing inappropriate mealtime behaviors. The accuracy of data collection by parents, the generalization of data on the consumption of non-target foods by the child, and the maintenance of results during follow-up evaluations were also assessed.
Thorsteinsdottir et al. (2022) [71]	Taste education	7 weeks; 2 parent sessions of 2 h; 6 cooking sessions of 90 min	Overall, the results suggest that the taste education program is promising as a brief, non-invasive, simple, and effective intervention. The findings also indicate that taste education methods can reduce parental concerns about children’s diets by providing simple tools. Further studies are warranted to explore these promising results (*p* = 0.002).
Chung et al. (2020) [42]	N/A	4 weeks; 3 sessions/week of about 1 h	Acceptance and habitual consumption of fruits and vegetables (fv) were assessed pre- and post-intervention. Results showed an increase in the consumption of fresh foods among children with ASD, with a significant increase only for bananas (from 55.3 to 78.0 g, *p* < 0.05). Physical changes to food may improve sensory processing in children with ASD, promoting fv acceptance and increasing habitual fv consumption.
Zhou et al. (2023) [43]	N/A	N/A; (N/A); (N/A)	The intervention reduced food-stealing episodes through self-control techniques, such as replacement behaviors using say-do correspondence training. Findings support this training as an effective, educational, and socially acceptable solution to food stealing in children with ASD and other developmental disorders. This study extends the applicability of say-do correspondence training to address problematic behaviors in community settings.
Davis et al. (2023) [44]	Simultaneous and sequential presentation	N/A; 2–4 sessions/day for 3–4 days/week of 10 min	Results showed the effectiveness of sequential presentation for preferred and non-preferred foods without requiring escape extinction techniques. Further research comparing sequential versus simultaneous food presentation is needed due to limited direct comparisons and mixed findings on their relative effectiveness.
Kral et al. (2023) [72]	M-health	3 months; (N/A); (N/A)	While no significant group-by-time interactions were found for primary outcomes (*p* > 0.25), there was a significant main effect of time for fv intake (*p* = 0.04), with both groups consuming more fv at 3 months (2.58 ± 0.30 servings/day) compared to baseline (2.17 ± 0.28 servings/day, *p* = 0.03). Children with low baseline fv intake and high technology engagement increased fv intake by 1.5 servings/day (*p* < 0.01). The intervention did not yield significant group differences, but future research should expand its impact across a broader range of foods and children with ASD.
Buro et al. (2023) [62]	Balance	8 weeks; 1 session/week; virtual mode of 15–40 min, focus group: 12 sessions of 45 min	The virtual balance intervention was well-received, with adolescents and parents reporting comfort with the format and interactive group setting. Participants noted improvements in psychosocial determinants of dietary intake, including knowledge and self-efficacy (*p* < 0.001). Further quantitative research is needed to examine behavioral outcomes.
Buro et al. (2022) [35]	Balance	8 weeks; 1 session/week of 45 min	Average sugar intake decreased (*p* = 0.026), and behavioral strategies (*p* = 0.010), self-efficacy (*p* < 0.001), and outcome expectations (*p* = 0.009) improved. However, no changes were observed in fv intake or other psychosocial determinants. The balance intervention may improve dietary behaviors in adolescents and young adults with ASD.
Gray et al. (2022) [73]	Autism eats	5 months; 10 sessions/week (N/A)	A novel integrated approach within early intervention (ei) services mitigated mealtime problems and promoted healthy eating habits in children with ASD. The “autism eats” program provides a unique opportunity to support young children and their families through sustainable interventions.
Manzanarez et al. (2021) [36]	N/A	8 months; 6 sessions/week of 90 min	Among the 50 participants, 38% attended initial sessions, and 26% completed the program. Families and staff expressed satisfaction, with parents reporting increased physical activity and fv intake in their children. Family-centered nutritional programs may positively influence eating behaviors in children with ASD and provide important insights for evidence-based practices.
Khan et al. (2021) [45]	N/A	1.5 years; 10 total sessions, 2/week of 20–30 min	Post-intervention results using the “com deal” oral-motor scale showed reduced problem scores and improved oral-motor skills. Structured and continuous oral-motor therapy also enhanced parents’ understanding of feeding difficulties in children with ASD.
Ivy et al. (2022) [58]	N/A	2 months; (N/A); 4 min	During baseline sessions, vegetable consumption was low but following the implementation of pre-meal presentation (pmp), consumption increased in 9 out of 16 participants. Pmp is a simple, cost-effective procedure suitable for group settings over extended periods.
Taylor et al. (2021) [16]	N/A	2–4 weeks; 6 sessions/week (average 11 sessions) of 7–8 h	All children met the therapeutic goals set at the start of treatment, consuming an average of 92 different foods and improving their eating and drinking behaviors. Clinically and statistically significant differences were maintained over a 2.3-year follow-up. Parents trained during the program successfully continued treatment at home.
Clark et al. (2020) [31]	Video modeling	8–10 weeks; 2 sessions/week of 1 h	Written instructions, video modeling, in vivo prompts, and feedback effectively trained parents to implement a structured meal procedure for addressing food selectivity in children with ASD. These findings suggest that clinicians could start training with written and video models, providing in vivo prompts and feedback as needed.
O’Connor et al. (2020) [19]	Video modeling	N/A; 3 sessions/day, 3 days/week of 1–10 min with 3–10 min breaks	Video modeling (vm) combined with differential reinforcement (dr) exposure increased food consumption. Direct exposure to reinforcement was necessary for effective intervention. Participants improved in food acceptance and consumption, meeting mastery criteria for target foods.
Matheson et al. (2019) [37]	Team-up	N/A; 16 total sessions of 1 h	Nearly two-thirds of participants attended at least 80% of treatment sessions. Parents reported high satisfaction, and children showed weight loss, increased physical activity, and vegetable consumption. Pilot data support the initial efficacy of the team-up program.
Kim et al. (2018) [46]	N/A	6 months; 4 sessions/week for a total of 96 of 5–10 min	Significant differences were observed in vegetable consumption (*p* < 0.05), but not in overall nutritional intake during regular meals.
Muldoon and Cosbey (2018) [64]	Eat-up	6 months; 3 sessions of 50 min	All children showed increased food acceptance and diversity, with reduced behavioral difficulties. Caregivers reported decreased problem behaviors during meals. Procedural fidelity improved among behavioral technicians and parents during the eat-up intervention.
Cosbey and Muldoon (2017) [59]	Eat-up	5 months; 5–6 base sessions, 9–21 coaching sessions (N/A)	All children demonstrated increased food acceptance (effect size > 0.90) and dietary diversity, with reduced problem behaviors.
Johnson et al. (2015) [38]	Pt-f	16 weeks; 9 sessions of 60–90 min	Results support the feasibility and initial effectiveness of the PT-F program for children with ASD and feeding issues.
Levin et al. (2014) [55]	N/A	N/A; 2–5 sessions/day of 30–45 min	Findings highlight the success of the “packaging” treatment for children with ASD and feeding difficulties.
Penrod et al. (2012) [56]	N/A	N/A; 10 and 14 sessions of 12–15 min	The intervention was implemented without escaping extinction and effectively increased food consumption in both participants.
Koegel et al. (2012) [17]	N/A	22 weeks; 4 sessions/week of 1 h	The study demonstrated increased flexibility and willingness to try new foods in response to an intervention combining individualized reinforcement with hierarchical food exposure.
Seiverling et al. (2012) [18]	N/A	N/A; about 20 tasting sessions/day, 1 meal every 10 sessions of about 10 min	Parent training improved parental performance, while children showed increased food acceptance and decreased disruptive behaviors. Parents reported an increase in the number of foods their children ate after the intervention and positively evaluated the training and feeding intervention.
